# ProteINSIDE to Easily Investigate Proteomics Data from Ruminants: Application to Mine Proteome of Adipose and Muscle Tissues in Bovine Foetuses

**DOI:** 10.1371/journal.pone.0128086

**Published:** 2015-05-22

**Authors:** Nicolas Kaspric, Brigitte Picard, Matthieu Reichstadt, Jérémy Tournayre, Muriel Bonnet

**Affiliations:** 1 UMR1213 Herbivores, INRA, Saint-Genès-Champanelle, France; 2 UMR1213 Herbivores, Clermont Université, VetAgro Sup, Clermont-Ferrand, France; Swiss Institute of Bioinformatics, SWITZERLAND

## Abstract

Genomics experiments are widely acknowledged to produce a huge amount of data to be analysed. The challenge is to extract meaningful biological context for proteins or genes which is currently difficult because of the lack of an integrative workflow that hinders the efficiency and the robustness of data mining performed by biologists working on ruminants. Thus, we designed ProteINSIDE, a free web service (www.proteinside.org) that (I) provides an overview of the biological information stored in public databases or provided by annotations according to the Gene Ontology, (II) predicts proteins that are secreted to search for proteins that mediate signalisation between cells or tissues, and (III) analyses protein-protein interactions to identify proteins contributing to a process or to visualize functional pathways. Using lists of proteins or genes as a unique input, ProteINSIDE is an original all-in-one tool that merges data from these searches to present a fast overview and integrative analysis of genomic and proteomic data from Bovine, Ovine, Caprine, Human, Rat, and Murine species. ProteINSIDE was bench tested with 1000 proteins identifiers from each species by comparison with DAVID, BioMyn, AgBase, PrediSi, and Phobius. Compared to DAVID or BioMyn, identifications and annotations provided by ProteINSIDE were similar from monogastric proteins but more numerous and relevant for ruminants proteins. ProteINSIDE, thanks to SignalP, listed less proteins potentially secreted with a signal peptide than PrediSi and Phobius, in agreement with the low false positive rate of SignalP. In addition ProteINSIDE is the only resource that predicts proteins secreted by cellular processes that do not involve a signal peptide. Lastly, we reported the usefulness of ProteINSIDE to bring new biological hypotheses of research from proteomics data: the biological meaning of the uptake of adiponectin by the foetal muscle and a role for autophagy during ontogenesis of adipose and muscle tissues.

## Introduction

A main challenge for scientists working on the efficiency of ruminant production and the quality of their products (meat, milk…) is to understand which genes and proteins control nutrient metabolism and partitioning between tissues or which genes and proteins control tissues growth and physiology [1–3]. These researches have produced vast amount of proteomics and genomics data and have used bioinformatics analyses to extract meaningful biological context for proteins or genes in ruminants [4–10]. Therefore, available bioinformatics tools implemented as web services are used to address these issues such as Amigo [11], Gorilla [12], or QuickGO [13] dedicated to Gene Ontology (GO) annotation, GeneCards [14] for an overview of the current available information about a gene or a protein for Human species, MiMi [15], IntAct [16], BioGrid [17], or PsicQuick [18] for protein-protein interactions (PPi) identification, STRING [19] or Apid2Net [20] for PPI identification and visualization as networks, and Sigcleave [21], SignalBlast [22], or SignalP [23] for the prediction of signal peptides. To gain insight efficiency and robustness, free and commercial tools such as DAVID [24], BioMyn [25], ToppGene [26], Expander [27], PathwayStudio [28], or the web-based software package Ingenuity Pathway Analysis (http://www.ingenuity.com; Redwood City, CA, USA) were developed as one-stop portal in order to combine two or three analyses, mainly GO annotations, PPI interaction, and information retrieval. Most of them do not permit to address data mining from genomic and proteomic studies in ruminants, except AgBase [29], a curated genomic database providing functional annotations and information for agriculturally important species, among them bovine and ovine. None of these resources include the prediction of proteins that are secreted outside the cell as recommended for a computational analysis of secretome [30]. However, in the context of livestock production, the efficiency of animals or the quality of their products not only depend on the functioning of a tissue but also on the interactions between tissues or cell types within a tissue [1], partly mediated by secreted proteins. Secreted proteins play important roles in tissue growth and homeostasis, as well as in tissues cross-talk and nutrient partitioning [2,31].

Here, we present ProteINSIDE, which aims to seamlessly integrate complementary analyses to produce information about proteins in one online automated package. ProteINSIDE comprises four modules: biological knowledge retrieval, annotations relative to biological process, molecular function, and subcellular location according to the GO, prediction of secreted proteins, and PPi analysed as a network. ProteINSIDE provides graphical and interactive results viewable on the website or downloadable. ProteINSIDE extracts information for a list of genes or proteins ID from myriad data sources with a unique input. Thus, it will circumvent searching for the different ID required for queries with the multiple existing bioinformatics tools, and it will save analysis time for the biologists. We demonstrated the higher or similar performances of ProteINSIDE relatively to currently used web-services or dedicated resources by a bench test that has evaluated results from 1000 random proteins by species. The relevance of results produced by ProteINSIDE was also checked with data that were previously partly analysed [3–5]. Lastly, we report the new knowledge related to adipogenesis, myogenesis, and the balance between both processes, provided by mining dataset from bovine foetal muscle and adipose tissues (AT) thanks to ProteINSIDE.

## Materials and Methods

### 1 ProteINSIDE features

**Fig 1:**
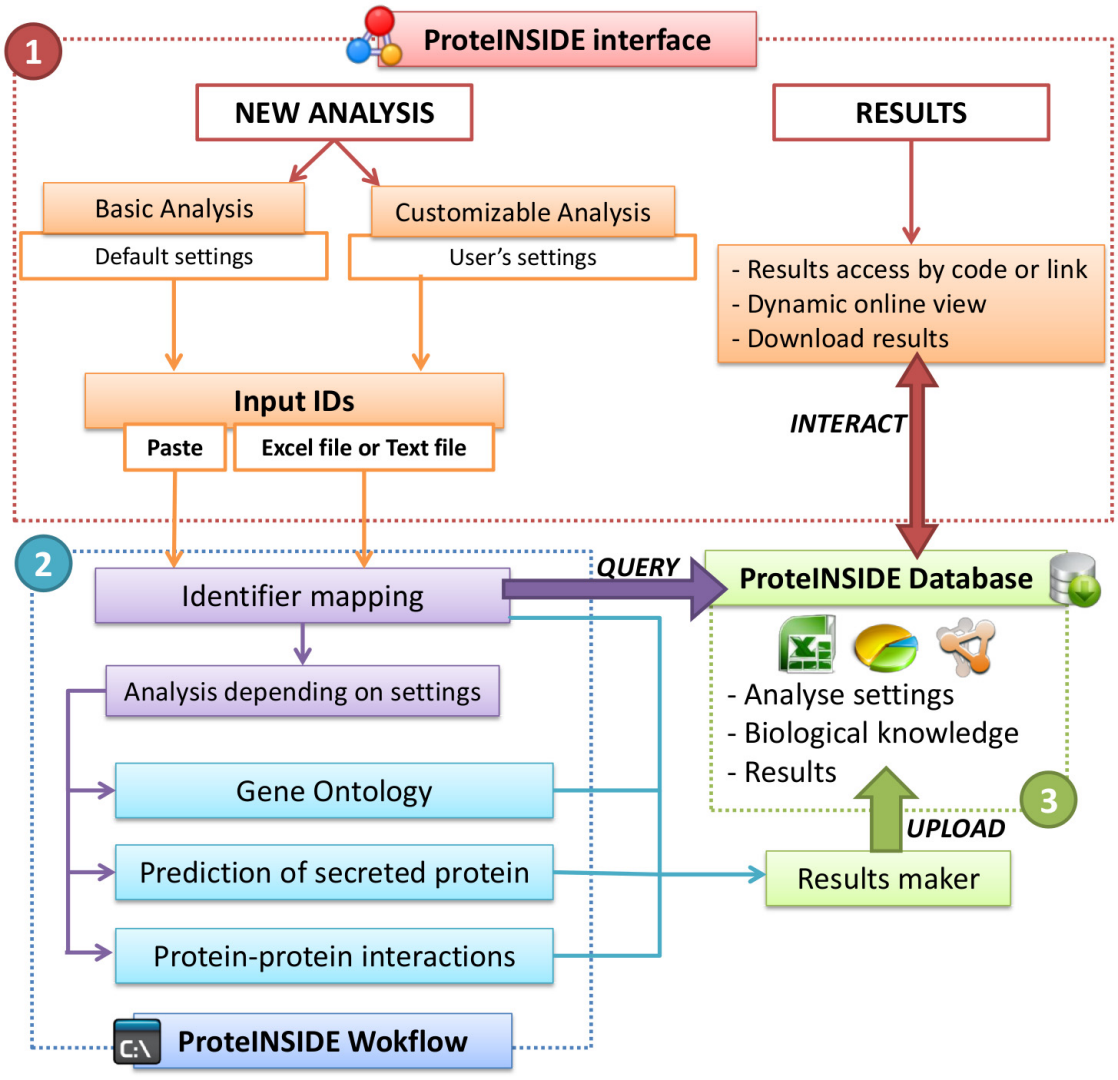
Flowchart of ProteINSIDE structure. The four modules to query the available biological information, annotate according to the GO, predict secreted proteins and visualize PPi, are either all run in the basic analysis or individually selected and run with specific settings in the custom analysis. The basic analysis runs ProteINSIDE with automatic settings. The custom analysis operates with the settings selected by the user: option to include GO Inferred from IEA codes (electronic annotation that are automatically unselected in the basic analysis), option to make GOTree chart networks with Cytoscape web, option to search PPi among the 31 databases proposed by ProteINSIDE, option to search PPi in other species using orthologous proteins, option to extend the PPi network with proteins that are not in the dataset, and option to choose the sensitivity to detect signal peptides with SignalP 4.1.

ProteINSIDE is an online workflow with an interface devoted to user-friendly and fully customisable analyses from lists of proteins or genes ID. Registered users have access to a private session to run, save, and visualise their results. Unregistered users can use ProteINSIDE, there is no analyses manager and results are deleted each month. Uploaded data are encrypted to ensure confidentiality. ProteINSIDE is divided into three parts: the workflow, the database, and the web interface. The workflow is a combination of Perl and R scripts to query databases, recover protein data, perform calculations, and run algorithms for signal peptide predictions and network visualisation. The MySQL database aims to reduce server load and stores settings and results from queries. ProteINSIDE’s database also stores available knowledge from major public biological databases. The web interface is the structure of ProteINSIDE and allows creating an analysis, viewing results, and keeping users informed with updates (Fig 1).

### 2 Setting up a basic or a custom analysis/query

ProteINSIDE performs either a “basic analysis” (in which settings are locked and the workflow acquires GO terms, signal peptide prediction, and PPi data from IntAct, UniProt, and BioGrid respectively) or a “custom analysis” (in which the user chooses the modules, the settings, and databases for PPi) (Fig 1). Previously defined settings for a custom analysis can be used for new queries by registered users using the “preset analysis”. To submit a basic or a custom analysis, users either directly paste a list of proteins or genes ID, or upload a file. Inputs can be protein (e.g., ADIPO_HUMAN) or gene name (e.g., ADIPO), protein accession number (e.g., Q15848), or also gene identifier (e.g., gi|62022275) from six species: Bovine, Human, Rat, Mouse, Ovine, and Caprine. A new analysis is run directly or is placed on a waiting list if the workflow is overloaded.

#### 2.1 Biological knowledge retrieval

Submitted ID, names, or accession numbers are compared to the ProteINSIDE database to ascertain a match with genes or proteins from Human, Rat, Murine, Bovine, Ovine, or Caprine species. This is achieved by merging the local database, which is updated every month, with data from UniProt [32] and NCBI (Gene, Proteins, and HomoloGene; [33]) public databases for all known genes and proteins from these six species.

#### 2.2 Functional annotation according to GO

ProteINSIDE imports GO terms by querying the QuickGO database. QuickGO was chosen because of its daily update, accessibility and performances. In the basic analysis, ProteINSIDE only imports GO terms that have been selected by evidence codes (GO terms Inferred from Electronic Annotation (IEA) are excluded) and agreed by curator review. The use of IEA is a setting of the custom analysis. ProteINSIDE provides the number of genes or proteins annotated by a specific GO term relatively to the total number of genes or proteins within both the dataset (frequency within the list) and the GO (frequency within the genome). The GO module of ProteINSIDE also analyses over- and under-represented terms to identify the most relevant and the most specific terms associated with the uploaded list, according to the functional enrichment first proposed by FatiGO [34]. Briefly, the frequency of a specific GO within the dataset is compared to its frequency in the genome. For this, ProteINSIDE prepares a 2 by 2 contingency table for a specific GO, performs a Fisher exact test and reports significant over-expressed GO based on the p-value of the test. The test is performed by an R script as follow:

mat <− matrix(c(A, B, C, D), nrow = 2)

tes <− fisher.test(mat)

where A is the number of proteins annotated by a specific GO term in the dataset, B the total number of proteins annotated in the dataset, C the number of proteins that are annotated by the specific GO term within the genome (also called “background of a GO term”), and D the total number of annotated proteins in the genome. ProteINSIDE provides the significantly over-expressed GO based on both unadjusted p-value of the test and a p-value corrected for multiple testing correction by the FDR Benjamini & Hochberg (BH) method [35].

The GO module also provides a view of network that links GO terms as a tree ancestor. The GO terms are linked by their parental association using ProteINSIDE database. ProteINSIDE database has its own version of ontology annotation from GO consortium [36] and QuickGO databases that relates: GO terms, biological functions, GO terms associations (relationships between terms only include “is_a” or “parts of” to select terms with a direct link), and the number of annotations by species. The “GOTree” view of ProteINSIDE is dynamic and only includes linked GO terms for a list of ID. The network is built with a custom version of Cytoscape web (v. 1.0.4) [37].

#### 2.3 Prediction of secreted proteins

In eukaryotes, at least five different routes of protein secretion out of the cells are reported: (I) the classical Golgi/ER-dependent secretory pathway for proteins that contain N-terminal signal peptide and non-classical protein exports or ER/Golgi-independent protein secretions that ensure protein secretion by (II) endosomal recycling, (III) plasma membrane transporter, (IV) membrane flip-flop, and (V) membrane blebbing that involves formation of vesicles or exosomes [38].

To identify proteins that are putatively secreted by the classical Golgi/ER-dependent secretory pathway, ProteINSIDE predicts the presence of a signal peptide on a protein sequence (imported by the biological knowledge retrieval script) through a local version of the SignalP tool (version 4.1 [23]). SignalP was chosen because of its high prediction score in comparison with other available tools [30,39] and the low false positive rate of approximately 6% (supplementary materials and methods of [23]). ProteINSIDE uses in the basic analysis the default cutoff value of 0.45 recommended by SignalP for a reliable prediction (optimized for correlation). A “sensitive” option is available in the custom analysis to increase the number of detected signal peptides (the cutoff value is decreased to 0.34), however with more false positive results (according to SignalP tutorial: http://www.cbs.dtu.dk/services/SignalP/performance.php). Then, ProteINSIDE checks that predicted secreted proteins are also annotated with GO terms related to secretion. For this purpose, we selected about 1200 GO terms related to secretion terms (monthly updated) as for example: secretion, vesicle, or also extracellular region. To identify proteins that are putatively secreted by non-classical protein exports or ER/Golgi-independent protein secretions, ProteINSIDE checks the subcellular location of proteins and compares their annotation relatively to our list of GO terms related to secretion. Lastly, to support these predictions (classical and non-classical ways) ProteINSIDE runs the TargetP software [40] that predicts the subcellular location of the proteins using the amino acid sequence. TargetP uses 4 cutoffs for its predictions: chloroplast (only for plant input), mitochondrion, secretory pathway (signal peptide), and other subcellular location. ProteINSIDE uses a preset cutoff option to get a significant prediction higher than 95% (according to TargetP tutorial: http://www.cbs.dtu.dk/services/TargetP/instructions.php).

#### 2.4 Protein-protein interactions and visualisation

PPi identification and visualisation within a network point out how various genes or proteins contribute to cellular or metabolic processes. ProteINSIDE uses Psicquic web service [18] to identify PPi and imports identified PPi by their “interaction detection methods” experimentally proven and agreed by curator review. Basic or custom analyses identify PPi within the uploaded dataset using the major preselected databases (IntAct, UniProt, and BioGrid chosen for the high data curation) or databases selected by the user (among 31 suggested by the PsicQuic web service). ProteINSIDE favours the use of multiple PPi databases in order to provide a more complete PPi network. In the custom analysis, ProteINSIDE identifies interactions between proteins from the dataset (core network) and proteins outside the dataset (extended network). ProteINSIDE identifies PPi recorded either within the species of the analysis or within a different species (to be selected among the six). To choose another species, user have either to select the Blastp [41] setting to know the similarity between the sequences of uploaded proteins and their orthologs, or to use conserved proteins declared in the HomoloGene database (only for Human, Bovine, Mouse, and Rat). For the extended network, ProteINSIDE does not include links between proteins outside of the dataset themselves in order to not overload the network visualization with too many interactions. ProteINSIDE focuses only on PPi between proteins of the dataset and other proteins of the analysis species. If users wish to view and analyze a complete network with links between proteins outside the uploaded dataset, they have to download the identifiers of the PPi extension (available on the PPi table results page) and run a new analysis.

Thus, ProteINSIDE constructs networks with PPi recorded in well- (Rat, Mouse, or Human) and poorly-annotated (Bovine, Ovine, or Caprine) species. The comparison of PPi networks allows expanding knowledge in the poorly- (Bovine, Ovine, and Caprine) relatively to well-annotated (Rat, Mouse, or Human) species. After a custom analysis with PPi extension, a GO analysis with the ID of genes/proteins of the extended network is directly launched by clicking on the button “Run a job to analyse the Gene Ontology for ID from this network”. Results of this GO analysis are available as a new work on the user home page, and provide GO terms with *p*-values for enrichment that take into account news values (related to the higher number of proteins) for A, B, C, D as defined in the GO annotation section 2.2. The PPi modules provide a view of proteins as networks which are built with Cytoscape web: edge colours inform on the experimental method used to identify the interaction. Each edge/experimental method has its own colour in the network and an interaction between two ID could have been detected by several experimental methods (this resulting in several edges between two ID). Key proteins can be highlighted using betweenness (that quantifies how frequently a node is on the shortest path between every pair of nodes for detecting bottlenecks in a network) or closeness (that quantifies how short are minimal paths from a given node to all others, a large closeness indicates that a node is close to the topological centre of the network) centralities as previously defined by Hwang et al. [42]. These centralities were used because they were proven as efficient to reveal key proteins that play important roles in a network [42].

#### 2.5 Results visualisation and download

**Fig 2:**
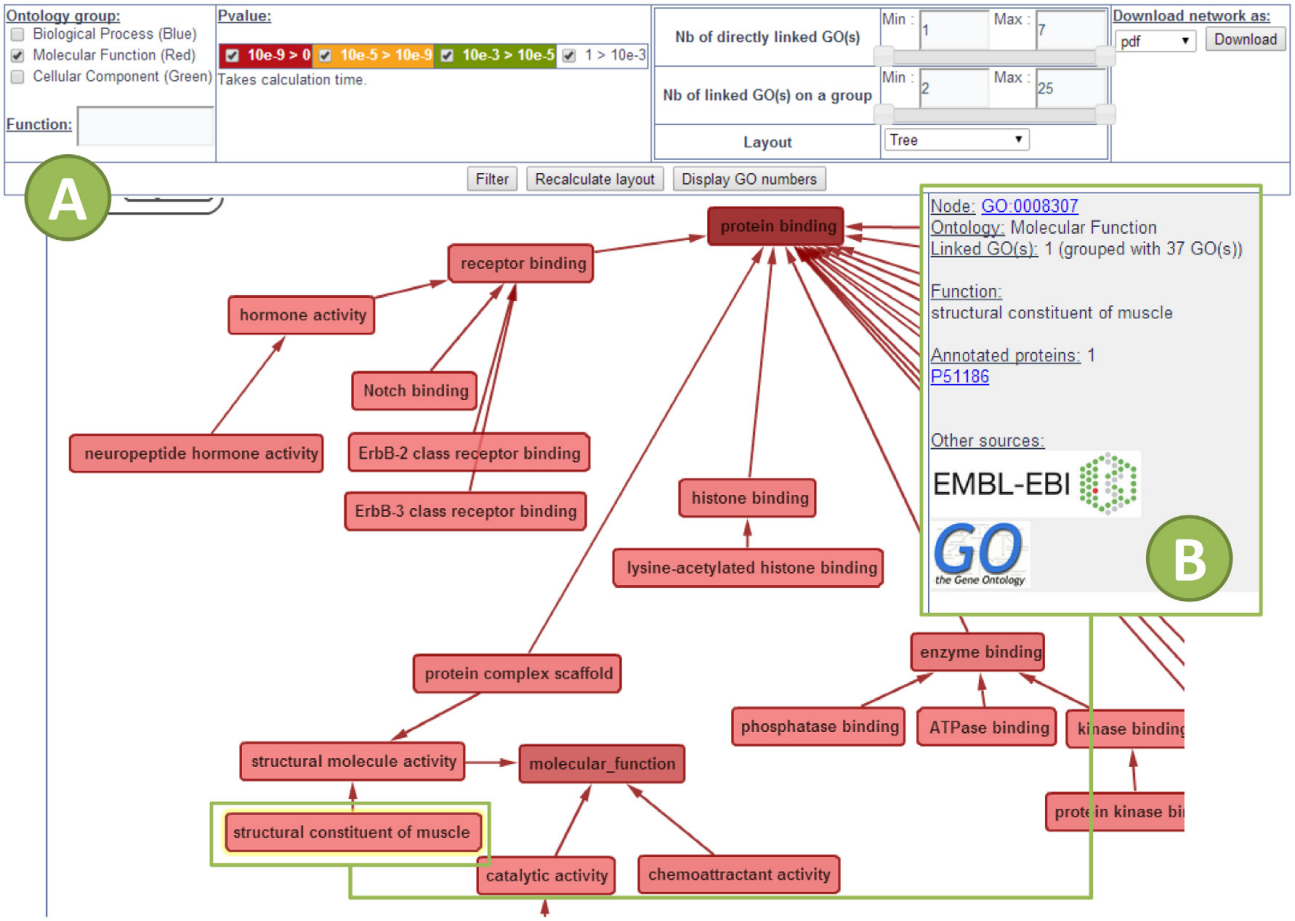
Dynamic GO tree network provided by ProteINSIDE. Network was built with Cytoscape web. Settings are available to sort the network according to Ontology groups, biological function, result of GO enrichment (p-value), numbers (Nb) of GO, or network layout. Go terms are sorted and colorized depending on the ontology group and the number of annotated proteins. (A) Dynamic network view of GO terms related to Molecular Function. (B) Clicking on a GO term provides the GO number, proteins from the sample list annotated by this GO, and links with public GO databases (AmiGO and QuickGO).

**Fig 3:**
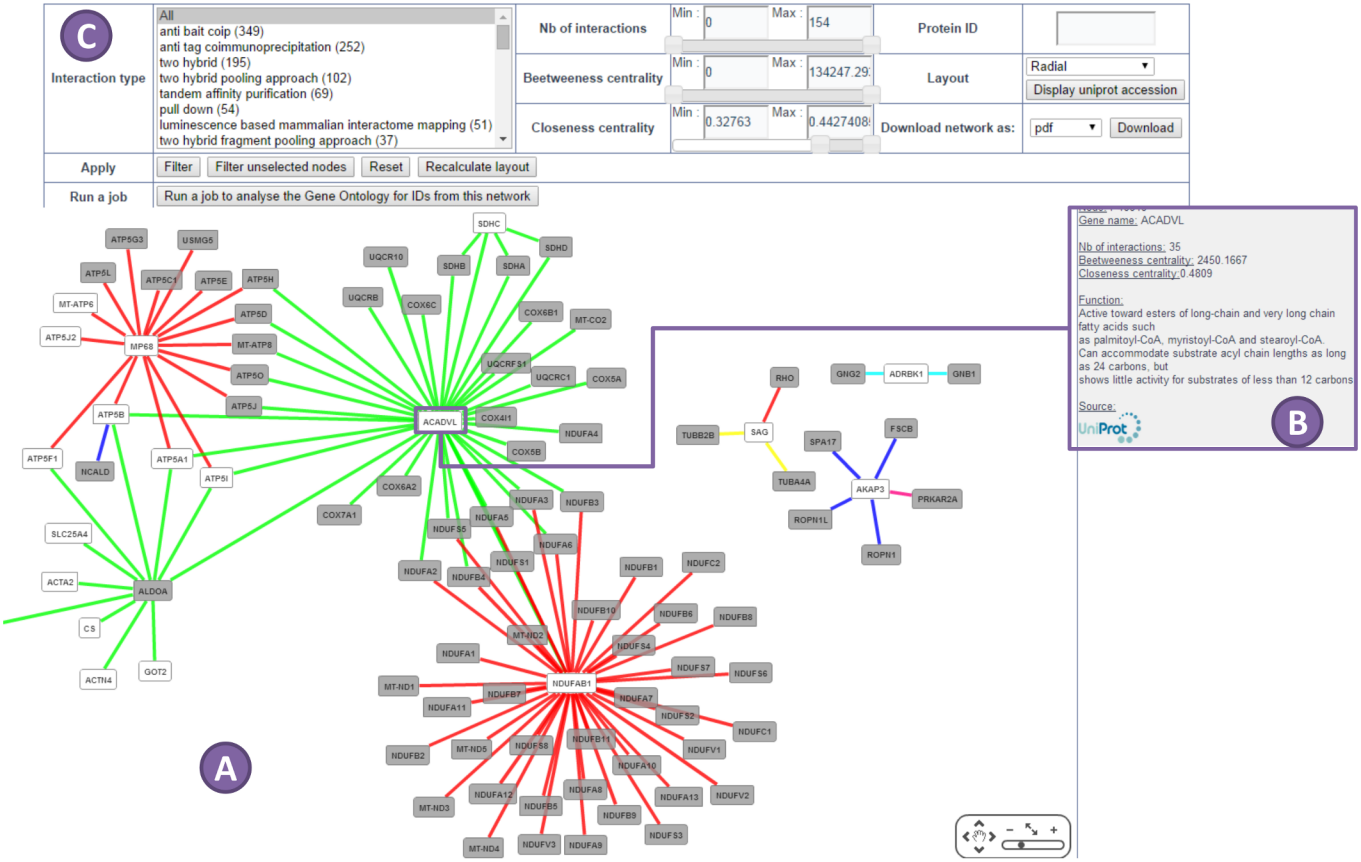
View of a PPi dynamic extended network made by ProteINSIDE. Network was built with Cytoscape web. (A) The colour of the edge depends on experimental methods used to identify PPi. White nodes are proteins from the dataset and grey nodes are known interacting proteins not included in the dataset. (B) Clicking on a protein/node provides biological information as gene and proteins ID, function, and a link to UniProt database. (C) Available options to sort the network and highlight proteins of interest.

Results are available through a unique code or a link provided after the submission of a list. Four separated pages provide results from the four analyses. The results are dynamic tables and charts that can be sorted and filtered online on the website by specific criterion such as biological function or protein and gene ID. Tables and charts are downloadable, and diagrams or histograms are printable. Networks are downloadable as picture (.pdf and. png) or as network software input (.sif or. xgmml or. graphml) files. Whatever the networks (GOTree or PPi), they are dynamics thanks to Cytoscape web which gives options to sort nodes, change layout, and search by proteins or biological function (Figs 2 and 3).

#### 2.6 Implementation

The web interface was programmed in PHP, HTML, and JavaScript. The workflow has been programmed using Perl (with CPAN modules (Comprehensive Perl Archive Network) and BioPerl [43]) and R scripts. The database was made in MySQL. By using these most common web dynamic programming languages, ProteINSIDE is completely available on any operating system using an internet browser (Internet Explorer, Firefox, Chrome, Safari…).

To ensure the sustainability of ProteINSIDE, we have set up an automatic update of the database. A program updates each month the biological information of the database of ProteINSIDE by comparison with the current and free NCBI and UniProt databases resources. Moreover, it gathers the information from the databases QuickGO and Amigo in order to update the GO term database of ProteINSIDE (function and annotated genes expected by GO term for each species) or to add new GO terms. The workflow update is done manually. If a program requires an update, it is set up and tested locally to avoid conflicts with running analyses of users. When the update is applied to ProteINSIDE: users can press the button "reload analysis" (green arrow) to restart previous analysis and to benefit to the new version of the workflow. Each new version of ProteINSIDE’s database or workflow is informed on the website by a new or by a message in the “About” section and the “User’s main page”. Users should be aware that results obtained with 2 different versions of ProteINSIDE may be not reproducible because of the deletion of obsolete data and the use of new ones.

### 3 Datasets

To test ProteINSIDE’s performances, we have created six datasets made of 1000 random proteins for each species (S1 Table lists the six samples files). These proteins have been selected using a script which queries randomly 1000 reviewed proteins for a species on the UniProt database. We have made separate custom analysis to test each part of the workflow using these datasets of 1000 random proteins by species. We have tested GO annotation using two customs analyses: with and without electronic annotation (option IEA). The prediction of secreted proteins has been bench tested using a custom analysis with SignalP prediction and GO annotation. Lastly, PPi were tested using four customs analyses: two analyses have queried the 3 databases (BioGrid, Uniprot, and IntAct) selected by default in the basic analysis to identify core and extended networks, and two analyses have queried 9 other databases (BioGrid, IntAct, MINT, MatrixDB, STRING, Reactome, InnateDB-IMEx, UniProt, and I2D-IMEx, among those to be chosen by the user) to identify core and extended networks.

Moreover, to demonstrate the added-value of the biological meanings provided by ProteINSIDE, we used 2 datasets that are lists of bovine proteins identified in the skeletal muscle *Semitendinosus* and the perirenal AT of Charolais and Blond d’Aquitaine foetuses [3–5]. The proteome was analysed at 60 (only for muscle), 110, 180, 210, and 260 days post conception (dpc) chosen according to previous data on muscle myogenesis [2]. As described in previous papers, all experimental procedures were approved by the Institutional Animal Care and Use Committee of INRA in accordance with the *Use of Vertebrates for Scientific Purposes Act* 1985. More recently the production of bovine foetuses was approved by the “Comité d’éthique en matière d’expérimentation animale Auvergne” (IACUC CEMEA Auvergne, agreement number CE 34–11).

## Results and Discussion

### 1 Evaluation of ProteINSIDE web service accuracy

We have tested ProteINSIDE with 1000 random proteins ID from six species by comparison with widely used resources (Table 1). DAVID [24] was chosen because it is a free and widely used resource (cited in more than 15500 publications, http://scholar.google.com/citations?user=dMn7gzYAAAAJ) in several species including the three ruminant species of interest, Bovine, Ovine, and Caprine. BioMyn [25] is a free comprehensive data warehouse that unifies, integrates and explores information related to Human genes and proteins. AgBase [29] is a curated warehouse of biological information and annotations specifically for species related to agriculture, among them Ovine and Bovine. We have compared results provided with three modules of ProteINSIDE to results from DAVID, BioMyn, and AgBase web services (Tables 2–4). To our knowledge, none of these three tools or current bioinformatics integrative resources performs the prediction of secreted proteins (Table 1). Results from the secretion module of ProteINSIDE were thus compared to two specific resources dedicated to the computational prediction of protein secretion PrediSi [44] and Phobius [45], chosen for their good scoring (supplementary data of [23] and [30]).

**Table 1:** Available modules of analyses for ProteINSIDE, DAVID, BioMyn, and AgBase.

Analyses	ProteINSIDE	DAVID	BioMyn	AgBase
**ID Mapping**	x	x	x	x
*Protein & Gene ID*	x	x	x	x
*Biological function*	x	x		x
*Chromosomal location*	x	x		
*Tissue specificity*	x			x
*Cellular location*	x			
**Gene Ontology**	x	x	x	x
*Functional annotation graph*	x	x	x	
*Enrichment tests*	x	x	x	
*Expected gene products*	x		x	
*GO dynamic network*	x			
*Select/unselect IEA*	x			x
*Database source*	x		x	x
**Prediction of secreted proteins**	x			
*Confirmed with GO of secretion*	x			
*Confirmed with TargetP prediction*	x			
**PPi**	x	x	x	
*Number of queried PPi databases*	31	4	10	
*Dynamic PPi network visualization*	x		x	
**Results**				
*Downloadable (Excel and/or text files)*	x	x	x	x
*Online interface to view and sort results*	x	x	x	
**ID conversion**	x	x		
**Orthologs conversion**	x			
**Species (*vs*. the 6 analysed by ProteINSIDE)**	6	6	Human	Bovine & Sheep
**Last Update *(frequency)***	Monthly	Sep. 2009	Mar. 2013	Monthly

Analyses performed by ProteINSIDE in comparison with DAVID [24], BioMyn [25], and AgBase [29]. ProteINSIDE performs analyses using gene or protein ID from 6 species: Human, Rat, Murine, Bovine, Ovine, and Caprine species.

x indicates that the analysis is performed by the resource.

**Table 2:** Comparison of identifier mapping results.

Species	ProteINSIDE	DAVID	BioMyn	AgBase
**Human**	1000	899	998	
**Mouse**	998	993		
**Rat**	1000	949		
**Sheep**	979	378		1000
**Goat**	1000	6		
**Bovine**	1000	959		1000
**% of retrieval for 6 species**	99.62	69.73		
**% of retrieval for ruminants (%)**	99.30^a^	44.77 ^a^		100 ^b^

Numbers of ID retrieved by the ID mapping module of ProteINSIDE and by DAVID, BioMyn, or AgBase when a list of 1000 random proteins per species was uploaded.

^a^ from Sheep, Goat and Bovine results

^b^ from Sheep and Bovine results

**Table 3:** Numbers of annotated proteins and GO terms provided by ProteINSIDE, DAVID, BioMyn, and AgBase.

Species		ProteINSIDE	DAVID	BioMyn	AgBase	Percent of results shared by ProteINSIDE and		
						DAVID	BioMyn	AgBase	
**Human**	Annotated proteins	816 (945)	- (864)	- (803)		- (90)	- (85)	
	GO terms	2676 (3641)	- (4167)	- (1930)		- (64)	- (50)	
**Mouse**	Annotated proteins	938 (997)	- (991)			- (89)		
	GO terms	2435 (3514)	- (3545)			- (55)		
**Rat**	Annotated proteins	783 (979)	- (950)			- (91)		
	GO terms	2790 (4074)	- (5134)			- (67)		
**Sheep**	Annotated proteins	159 (916)	- (372)		125 (938)	- (40)		78 (100)
	GO terms	834 (2446)	- (1252)		1069 (2541)	- (51)		100 (100)
**Goat**	Annotated proteins	32 (503)	- (6)			- (1)		
	GO terms	190 (612)	- (82)			- (4)		
**Bovine**	Annotated proteins	365 (886)	- (957)		286 (898)	- (86)		77 (100)
	GO terms	1418 (3085)	- (3850)		1569 (3130)	- (61)		100 (100)

The percent of results shared by ProteINSIDE and by DAVID, BioMyn, or AgBase were calculated for comparison.

() for annotations that include IEA (Inferred electronic annotation).

- indicates that the resource does not provide annotation without IEA.

**Table 4:** Number of PPi identified using ProteINSIDE.

	3 databases		9 databases	
	PPi within the dataset	PPi outside the dataset	PPi within the dataset	PPi outside the dataset
**Human**	396	5518	1703	6269
**Mouse**	111	1410	702	8475
**Rat**	27	271	42	425
**Sheep**	0	0	0	0
**Goat**	0	0	0	0
**Bovine**	12	96	34	131
**Bovine EXT Human**			12	5921

ProteINSIDE had queried both 3 (BioGrid, Uniprot, and IntAct selected by default in the basic analysis) and 9 (BioGrid, IntAct, MINT, MatrixDB, STRING, Reactome, InnateDB-IMEx, UniProt, and I2D-IMEx chosen by the user) databases to record PPi that have been identified by experiments. Within a species, PPi were searched between proteins within (core network) and outside (extended network) the dataset. Bovine proteins ID were uploaded to search for known interactions with their orthologs in Human (EXT Human).

#### 1.1 Biological information retrieval by mapping Identifiers: ID Resume

The high ability of ProteINSIDE to retrieve biological information for 1000 random proteins ID from Human, Rat, Murine, Bovine, Ovine, or Caprine species is shown by the retrieval of 100% of ID from Bovine, Goat, Human, and Rat; as well as of 99.8% and 97.8% of ID from Mouse and Sheep, respectively (Table 2). These ID retrievals by ProteINSIDE were similar to those of BioMyn for Human ID or AgBase for Ovine and Bovine ID, but higher than those of DAVID, especially for Ovine (38% of ID retrieved) and Caprine (0.1% of ID retrieved) species (Table 2). The differences between the performances of ProteINSIDE, BioMyn or AgBASE, and DAVID may come from the lack of a recent database update for DAVID and the consequent missing of biological information (Table 1).

For each uploaded ID, ProteINSIDE obtained and summarized, as a downloadable table, the gene or protein ID, gene and protein names, the protein function, the gene chromosomal location, information on tissues expression, the cellular location, orthologous ID, and the FASTA sequence of the protein. These results are directly viewed on the “ID resume” web page of ProteINSIDE, and each protein or gene ID are linked to corresponding UniProt and NCBI web pages. A part of these biological data is also provided by DAVID, BioMyn, and AgBase.

#### 1.2 Gene Ontology annotation

ProteINSIDE annotated proteins or genes ID with GO terms selected by evidence codes (IEA are excluded) and agreed by curator review in the basic analysis, and additionally with GO terms from IEA as a setting of the customs analysis. As ProteINSIDE, AgBase allowed unselecting IEA, while DAVID and BioMyn use IEA by default. Thus, results from the GO module of ProteINSIDE for 1000 random proteins ID were compared to DAVID and BioMyn results with IEA, and to AgBase results with and without IEA (Table 3).

On average 90% of proteins ID from Human, Rat, or Mouse annotated by ProteINSIDE were also annotated by DAVID. However, the percent of proteins annotated both by ProteINSIDE and DAVID decrease to 86%, 40%, and 0.6% for Bovine, Ovine, and Caprine ID, respectively. The numbers of GO terms from DAVID were around 52% higher for Human, Mouse, Rat, and Bovine proteins but 86% and 48% lower for Caprine and Ovine proteins, respectively, when compared to ProteINSIDE. Consequently, around 64% of GO terms from ProteINSIDE were common to those provided by DAVID for five species. On average, 85% of Human proteins were annotated both by ProteINSIDE and BioMyn with 50% of GO terms provided by the two resources. For Bovine and Ovine proteins, ProteINSIDE annotated more proteins with less GO terms than AgBase in analyses without IEA, while AgBase annotated more proteins with more GO terms when IEA annotations were used. In both analyses, GO terms provided by ProteINSIDE were all retrieved with AgBase. The annotation differences between ProteINSIDE and DAVID, or to a lesser extend BioMyn, may result from the lack of a recent update of the databases used. For example, from our lists of proteins, DAVID had provided GO terms that were declared obsolete by the GO consortium (GO:0006096 previous term was “Glycolysis”, replaced the 29^th^ march 2014 by “Glycolytic process”) that thus could not be retrieved by resources such as ProteINSIDE and AgBase (that integrate the GO consortium updates). It is noteworthy that AgBase provided reliable GO annotations for two ruminant species, probably because of their own curated database that is also monthly updated. Thus, both AgBase and ProteINSIDE perform GO annotations for bovine and ovine, while only ProteINSIDE annotates ID from Caprine species. It is noteworthy that ProteINSIDE annotated on average 45% more proteins with 46% more GO terms when using IEA by comparison without IEA.

The results are viewed on the “GO” web page of ProteINSIDE as tables, diagrams, and GOTree charts. Unlike the other available tools, ProteINSIDE summarized main results of GO as GOTree charts which are ordered tree layout networks that link related GO terms (Fig 2).

#### 1.3 Secreted proteins

**Fig 4:**
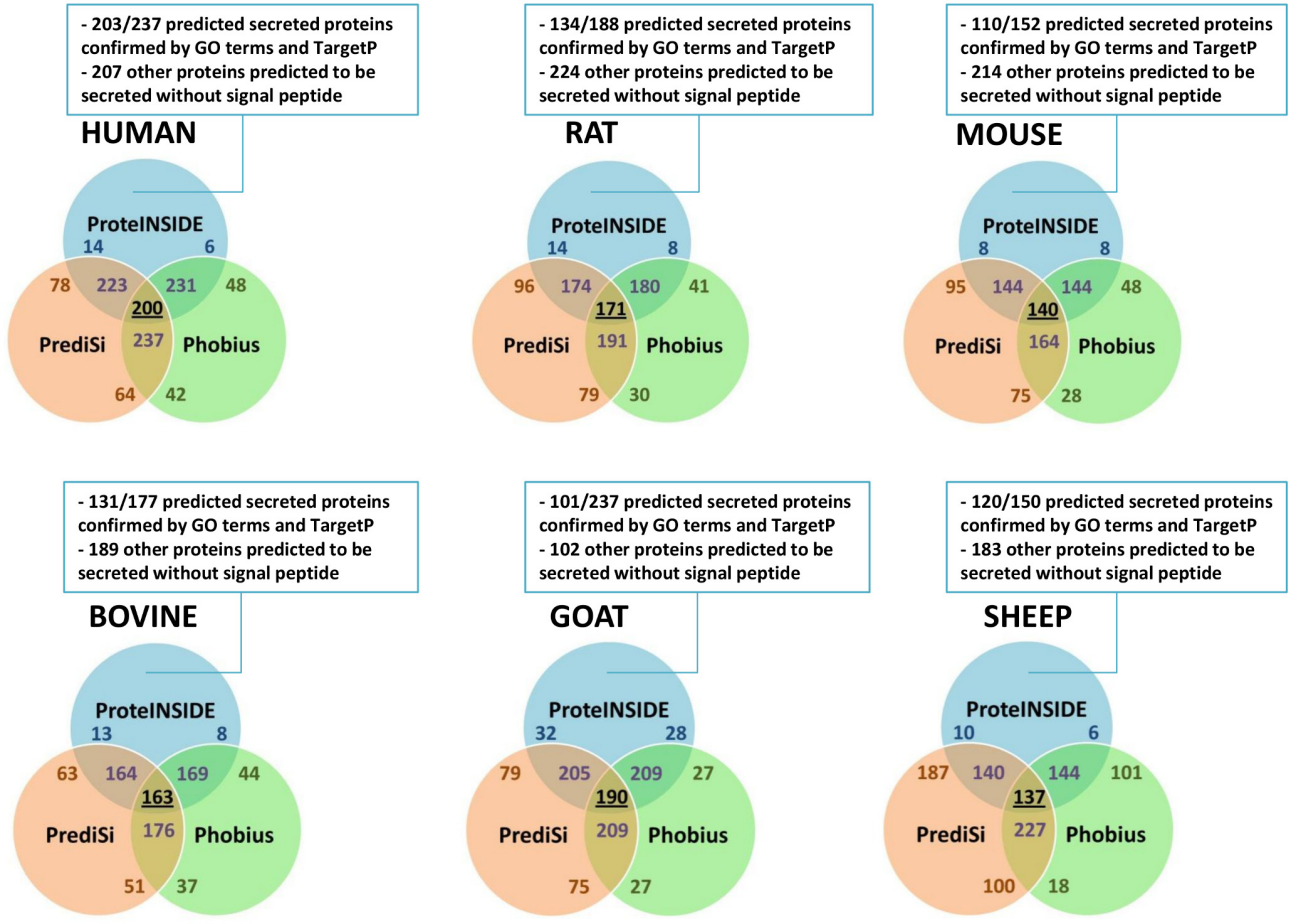
Venn diagrams of predicted signal peptides from amino acid sequence of 1000 proteins. Resources used to predict signal peptides were SignalP that was included in ProteINSIDE, as well as PrediSI [44] and Phobius [45] for bench tests. To reinforce the prediction of proteins to be secreted, ProteINSIDE checked the cellular localization of proteins with TargetP and used GO terms relative to the cellular component. GO terms were also used to predict proteins that are secreted without signal peptide. Squares give numbers of predicted (SignalP) secreted proteins confirmed by GO terms and subcellular location prediction (TargetP), and confirmed by GO terms for proteins that are potentially secreted without signal peptide.

Whatever the species, 95% of signal peptides predicted by ProteINSIDE thanks to SignalP were also predicted by PrediSi and Phobius (Fig 4) for 1000 random protein ID. However and as expected, the number of signal peptides predicted by ProteINSIDE was 49% and 24% lower than those predicted by PrediSi and Phobius, respectively. This may result from the known higher false positive rate of prediction by PrediSi and Phobius (52% and 16% according to [23], respectively) than SignalP 4.1 (6%). As an example of false positive, ALKBH5 was predicted as a secreted protein by Phobius and PrediSi but not by ProteINSIDE, while this is a nuclear protein according to UniProt database.

An added-value of ProteINSIDE is the use of GO terms and TargetP, both to reinforce the prediction of proteins that are secreted (Table 1). By adding these two methods, 48% (Fig 4) and 12% (in average for the six species, data not shown) of predicted secreted proteins with and without signal peptide were confirmed by GO terms and TargetP prediction, respectively. This two-step analysis improves the reliability of proteins proposed to be secreted. Moreover, unlike other available resources ProteINSIDE predicts proteins exported out of eukaryote cells by non-classical secretory pathways (without signal peptide). However, all of these predictions remain to be confirmed by new or already published experiments. Lastly, GO terms confirmation can be improved by the use of IEA codes for annotations.

Results are viewed on the “Secreted Protein” web page of ProteINSIDE as two main tables: the first table listed proteins secreted by the classical Golgi/ER-dependent secretory pathway thanks to a signal peptide and the second table listed proteins that are predicted to be secreted by non-classical secretory pathways.

#### 1.4 Protein-Protein interactions

The number of PPi identified by querying 3 major (UniProt, BioGrid, and IntAct preselected in the basic analysis) or 9 widely used PPi databases (BioGrid, IntAct, MINT, MatrixDB, String, Reactome, InnateDB, I2D, and UniProt, chosen thanks to the settings of the custom analysis) are summarized in Table 4. PPi were searched between proteins within (core network) and outside (extended network) the dataset. As expected, the number of PPi identified was higher when 9 rather than 3 databases were queried both for core and extended networks (except sheep and goat, Table 4). Martha and co-workers [46] have previously emphasized the need for a trade-off between the coverage and the reliability of PPi. The maximal coverage can be achieved as we have done by using a traditional union approach for PPi integration, which might however increase false positive PPi. We attempted to minimize the number of false positive by selecting PPi that have been experimentally detected only. With this caution, ProteINSIDE constructs networks with PPi from multiple public PPi databases in order to increase the recovery of species interactome: better informed network with more curated PPi. It is noteworthy that none PPi were identified between Ovine or Caprine ID, which is consistent with the lack of experiments that search for PPi in these species. However, ProteINSIDE database is monthly updated and latest experimentally proven PPi from these species would be identified in the future. Because PPi in ruminant remains poorly recorded, ProteINSIDE automatically constructs networks from ruminant ID within well-studied species chosen either in a blind way by the user or from results of alignments (Blastp) between the sequences of uploaded proteins and their orthologs. It may be useful for biologists working with ruminant species to identify the relationships among different proteins in order to understand the contribution of genes or proteins to pathways or cellular processes.

On the “Protein Interactions” web page of ProteINSIDE, PPi are viewed as tables of listed PPi and as dynamic networks. A dynamic network (Fig 3) links PPi depending on the detection method, the number of interactions by node, the high-centrality (closeness centrality) and the high-degree (betweenness centrality) nodes. To our knowledge, ProteINSIDE is original by the friendly view of networks thanks to the sorting options that provide opportunity to users to identify key nodes (with high closeness and betweenness centralities) both on core and extended networks.

The bench test had provided data in favour of the reliability and accuracy of results produced by ProteINSIDE. We have then tested the ability of ProteINSIDE to produce new hypotheses of research and knowledge for biologists.

### 2 Analysis of proteomics data from bovine adipose and muscle tissues using ProteINSIDE

Custom analyses were proceeded with 143 and 120 proteins ID identified by proteomics from perirenal AT [4] and *Semitendinosus* muscle [3,5] sampled from the same bovine foetuses at key developmental ages. With these datasets, we aimed firstly to check for the relevance of ProteINSIDE results and secondly to focus on new knowledge provided by ProteINSIDE by comparison with our previous analyses [3–5]. Settings used for custom analyses were: Gene Ontology with IEA, prediction of secreted proteins with default settings, and PPi research on 9 widely used databases (BioGrid, IntAct, MINT, MatrixDB, STRING, Reactome, InnateDB-IMEx, UniProt, and I2D-IMEx).

#### 2.1 Foetal Bovine AT features

ProteINSIDE successfully uploaded and provided a fast overview of the biological information available in UniProt and NCBI databases for 143 proteins that were identified in the AT at each foetal age.

To check for the relevance of ProteINSIDE results, we compared GO terms recovered by ProteINSIDE to those previously published after a GO analysis with DAVID [4]. We reported here enriched GO terms related to biological processes for proteins from clusters 1 (9 proteins highly abundant at 180 dpc [4]; S2 Table) and 8 (20 proteins highly abundant at 260 dpc [4]; S2 Table). These clusters were chosen for their very different expected GO annotations related to early and late steps of adipogenesis. In agreement with previous GO annotations [4], enriched GO terms recovered by ProteINSIDE were related to cytoskeleton organization, regulation of actin filament depolymerisation, and regulation of apoptotic process for proteins from cluster 1. GO terms recovered for cluster 8 were related to glycolytic process, tricarboxylic acide cycle, and oxidation-reduction processes. In addition for proteins from cluster 8, ProteINSIDE had identified new enriched GO terms such as lipid metabolic process (related to proteins THEM4, ECH1, ACAT2, ECHS1, GPD1, and PCCB) and fatty acid metabolic process (ECHS1, ECH1, PCCB, and THEM4) that are consistent with the highest adipocyte volume and total lipogenic activities in perirenal AT at 260 dpc [4]. These new and relevant annotations recovered by ProteINSIDE may result from a better annotation of bovine proteins taken into account by ProteINSIDE thanks to its regular update.

ProteINSIDE also brought new knowledge since it had predicted 18 proteins as secreted thanks to a signal peptide. Among them, 13 were also annotated by GO terms relative to secretion processes and predicted to be located out of the cell either by TargetP or the subcellular location provided by UniProt (Table 5). In agreement with our proposed secretome of foetal AT, SERPINA1, APOA1, APOA2, TTR, ALB, TF, HSP90B1, PDIA3, and ADIPOQ were identified in the proteome of bovine plasma [47]; AFP was assayed in the plasma of baboon foetuses [48]. The 3 remaining proteins were reported to be either located on plasma membrane or extracellular vesicular exosome (a membrane-bounded vesicle that is released into the extracellular region) for ERLIN2, in blood for FGG, or component of the extracellular matrix for COL6A2 [UniProt Sources]. The 5 proteins (NDUFS8, NDUFS3, NDUFA10, PCCB, and RCN1) predicted to be secreted but not confirmed by GO terms and TargetP were proven to be intracellular and located in the mitochondrion or endoplasmic reticulum [UniProt Sources]. They were listed as secreted because SignalP identified a signal peptide on their amino acid sequences for an intracellular rather than plasma membrane translocation (false positive). These results highlight the usefulness of the integration of results from both prediction and annotations for a reliable list of proteins proposed to be secreted. ProteINSIDE had also predicted 89 potentially secreted proteins without signal peptide. However, only 11 proteins were confirmed by GO terms, TargetP or known subcellular location in UniProt (PRDX6, MDH1, ALAD, VCP, CAPZA1, CAPZA2, TPI1, ARHGDIA, HNRNPK, EIF5A, and TPMT). Among them, 6 proteins are known to be secreted by AT (PDRX6 [49]; MDH1 and TPI1 [50]; VCP, ARHGDIA, and EIF5A [51]). Thus, by using ProteINSIDE, we have identified 13 proteins potentially secreted thanks to a signal peptide and 11 proteins potentially secreted by non-classical ways. It is noteworthy that these proteins could be synthesized by either AT such as adiponectin [4] or other tissues and taken up by AT, that remains to be questioned by the search of their mRNA in AT in available published results or by new experiments.

**Table 5:** Potentially secreted proteins with a signal peptide in bovine foetal AT, muscle or both tissues.

Adipose tissue	Muscle tissue	Adipose and muscle tissues
ADIPOQ ^1^ ^,^ ^2^ ^,^ ^3^	ADIPOQ ^1^ ^,^ ^2^ ^,^ ^3^	ADIPOQ
NDUS3	ALB ^1^ ^,^ ^2^ ^,^ ^3^	APOA1
ERLIN2 ^2^ ^,^ ^3^	AFP ^2^ ^,^ ^3^	AFP
NDUS8	APOA1 ^1^ ^,^ ^2^ ^,^ ^3^	ALB
NDUFA10	GSN ^1^ ^,^ ^2^ ^,^ ^3^	PDIA3
SERPINA1 ^1^ ^,^ ^2^ ^,^ ^3^	GARS ^1^	SERPINA1
APOA1 ^1^ ^,^ ^2^ ^,^ ^3^	SERPINA1 ^1^ ^,^ ^2^ ^,^ ^3^	
APOA2 ^1^ ^,^ ^2^ ^,^ ^3^	P4HB ^1^ ^,^ ^2^ ^,^ ^3^	
FGG ^1^ ^,^ ^2^ ^,^ ^3^	PDIA3 ^1^ ^,^ ^2^	
TTR ^1^ ^,^ ^2^ ^,^ ^3^		
ALB ^1^ ^,^ ^2^ ^,^ ^3^		
AFP ^1^ ^,^ ^3^		
TF ^1^ ^,^ ^2^ ^,^ ^3^		
PCCB		
COL6A2 ^1^ ^,^ ^2^ ^,^ ^3^		
HSP90B1 ^2^ ^,^ ^3^		
PDIA3 ^2^ ^,^ ^3^		
RCN1		

^1^ confirmed by GO terms

^2^ confirmed by TargetP

^3^ confirmed by Subcellular location provided by UniProt resource

**Fig 5:**
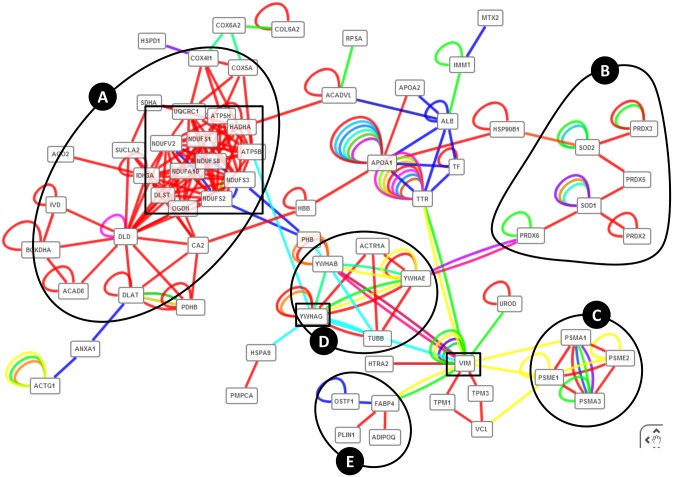
Interactions network between proteins of the bovine foetal AT. We highlighted the relationships among different proteins involved in a same process: (A) mitochondrial metabolism, (B) redox activity, (C) proteasome complex, (D) cell proliferation, and (E) differentiation and metabolism of AT. Squares indicate key proteins identified by sorting the network with algorithms of betweeness (an average of 400) and closeness centralities (an average of 0.3).

The PPi module of ProteINSIDE had revealed that 107 proteins of the dataset were linked by 300 interactions (Fig 5). The synthetic view of PPi as network had shown 5 subnetworks of proteins contributing to a same process: cell proliferation (Fig 5D), proteasome complex (Fig 5C), complex I and III of the respiratory chain (Fig 5A), redox activity (Fig 5B), and differentiation and metabolism of AT (Fig 5E). Then, to identify key proteins in the foetal AT growth we have sorted the network by applying high values of betweenness and closeness centralities and we proposed 15 proteins as centrals in the network (Fig 5). Among them, 13 were mitochondrial proteins involved in Krebs cycle, respiratory chain, and branched chain amino acid metabolism, in agreement with metabolic pathways that were proposed to control the increase in foetal adipocyte size in our previous analysis [4]. The last 2 keys proteins were YWHAG previously proposed to balance cell cycle progression and cell cycle arrest that are two major cellular events for adipocyte precursor proliferation and differentiation, as well as Vimentin (VIM) that was not identified as key protein in our previous data mining [4]. However, VIM was reported to be over-abundant at 110 and 180 dpc or with a stable abundance across foetal ages depending on the isoforms. VIM, a class-III intermediate filament found in various non-epithelial cells, especially mesenchymal cells was first proposed to sustain the well-described dramatic change in cell shape from fusiform adipocyte precursors to spherical adipocytes [4] during adipogenesis. More recent results have shown that VIM contributes to both the formation of lipid droplets through an interaction with perilipin [52] and the regulation of fatty acid transport through an interaction with FABP4 [53]. Thus, the central role of VIM in the foetal growth of AT proposed by ProteINSIDE is sustained by experimental results that emphasized its role in early and late stages of adipogenesis.

#### 2.2 Foetal Bovine muscle tissue features

ProteINSIDE correctly identified 120 muscular proteins present at all the five foetal ages [3,5] and provided a fast overview of biological information available in UniProt and NCBI databases.

As for data from AT, the relevance of ProteINSIDE results was checked. We have compared enriched GO terms recovered by ProteINSIDE to those previously published [3]. We reported here enriched GO terms related to biological processes for proteins from cluster 8 (18 proteins over-abundant at 60 dpc [3,5]; S2 Table) and cluster 1 (14 proteins over-abundant at 180 dpc [3,5]; S2 Table). In agreement with previous GO annotations [3,5], enriched GO terms recovered by ProteINSIDE were related to the negative regulation of apoptotic process, DNA binding, and skeletal muscle thin filament assembly for cluster 8, and to glycolytic process and the regulation of translational initiation for cluster 1. These GO related to cellular and molecular events reflect the proliferation of muscular cells before 180 dpc and, their metabolic differentiations, after in accordance with the know pivotal role of the foetal age 180 dpc [6].

As new knowledge, ProteINSIDE had predicted 9 secreted proteins among the 120 proteins from foetal muscle (Table 5). Among them, 7 proteins were confirmed by TargetP, subcellular location in UniProt, and GO terms related to secretory pathways while PDIA3 and GARS were only confirmed by one GO term. Available data from proteomics or specific isolations in bovine [47], human [54,55] and baboon [48] plasma confirm that APOA1, P4HB, GSN, ADIPOQ, AFP, ALB, PDIA3, and SERPINA1 are secreted proteins. The protein GARS was a false positive of the ProteINSIDE prediction since available experimental data recorded in UniProt have located this protein in the cytosol or the mitochondrion [UniProt sources: http://www.uniprot.org/uniprot/P41250]. ProteINSIDE had also predicted 73 proteins secreted by cellular pathways that do not involved signal peptide. However, 11 proteins (F3, CAPZA2, TPI1, ARHGDIA, HNRNPK, EIF5A, SNX6, ANXA2, COPS4, PRDX6, and AKR1B10) were confirmed by TargetP, GO terms or subcellular location in UniProt. Among them, 6 proteins are known to be secreted by skeletal muscle (TPI1, ARHGDIA, HNRNPK, EIF5A, PRDX6 [56], and ANXA2 [57]). Thus, with ProteINSIDE we have identified 8 and 11 proteins potentially secreted by a signal peptide or by other secretory pathways, respectively. As for AT, the remaining question is whether these proteins are synthesised or taken up from the blood by the muscle. Available results from mRNA tissue-distribution show that AFP is synthesized by the liver and taken up by the muscle, since no mRNA was detected in this tissue [48]. The synthesis of ADIPOQ (originally thought to be limited to adipocytes) by muscular cells remains controversial because of the very low mRNA abundance in L6 myotubes when compared to white AT [58] and, its immunohistochemical location in vascular endothelium and adipocytes of the gastrocnemius muscle [59].

**Fig 6:**
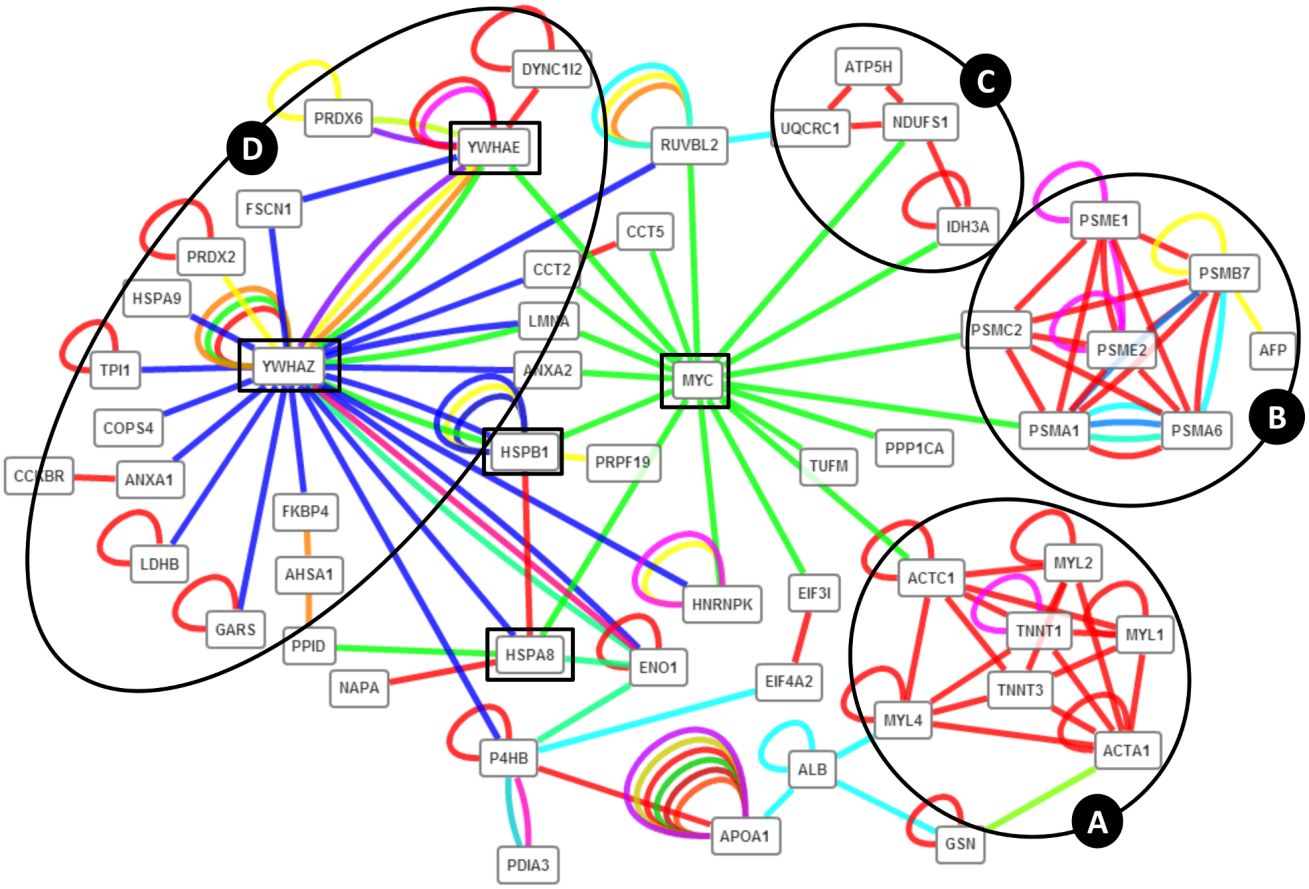
PPi network between proteins from the bovine foetal muscle. We highlighted the relationships among different proteins involved in a same process: (A) muscle development, (B) energetic complexes, (C) respiratory chain, and (D) cell proliferation. Squares indicate key proteins identified by sorting the network with algorithms of betweeness (an average of 100) and closeness centralities (an average of 0.4).

ProteINSIDE revealed 171 interactions between 76 proteins of the dataset (Fig 6). A synthetic view of PPi as network had shown 4 subnetworks of proteins contributing to a same process: the muscle development (Fig 6A), cell proliferation (Fig 6D), energetic complex (Fig 6B), and respiratory chain (Fig 6C) in agreement with the previous pathways proposed to sustain muscle growth in bovine foetuses [3,5]. Using high levels of betweenness and closeness centralities, we proposed for the first time 5 proteins as central in this network and for foetal muscle growth (square in Fig 6): YWHAZ and YWHAE regulating the balance between cell cycle progression and apoptosis, MYC an activator of the transcription of growth-related genes, and HSPB1 and HSPA8 involved in the protection of structural proteins (like desmin, actin, and titin) and also known as anti-apoptotic factors, promoting cell survival. Interestingly, HSPB1 was identified as a central protein in a network of protein related to muscle hypertrophy and functioning in growing bovine [60]. Moreover, HSPB1 was proposed to regulate the total number of fibres in Bovine foetuses (B. Picard, unpublished data). Present results suggest that HSPB1 may also contribute to myogenesis that is sustained by a very recent study reporting a repressed formation of myotubes by siRNA inhibition of HSPB1 expression in bovine myogenic cell, while expression of HSPB1 enhanced the expression of desmin and accelerated formation of myotubes [61].

#### 2.3 An union analysis of adipose and muscular data to identify pathways involved in the growth of AT and muscle and their cross-talk

There is striking evidence for developmental and functional links between muscle and AT: the successive waves of growth of muscle and AT suggest a priority for muscle growth, and the comparison between lean and fat bovine breeds suggest that an increased muscular development is concomitant with a decrease in AT mass [2]. In order to increase our knowledge on pathways involved in the growth of both AT and muscle and in the cross-talk between them, we have compared the adipose and muscular datasets. We have identified 46 proteins present both in adipose and muscle tissues that were subjected to a custom analysis with the following settings: GO annotation without IEA codes, prediction of secreted proteins, PPi research on 9 widely used databases (BioGrid, IntAct, MINT, MatrixDB, STRING, Reactome, InnateDB-IMEx, UniProt, and I2D-IMEx), and an extended network of PPi with proteins outside of the dataset in Human.

**Fig 7:**
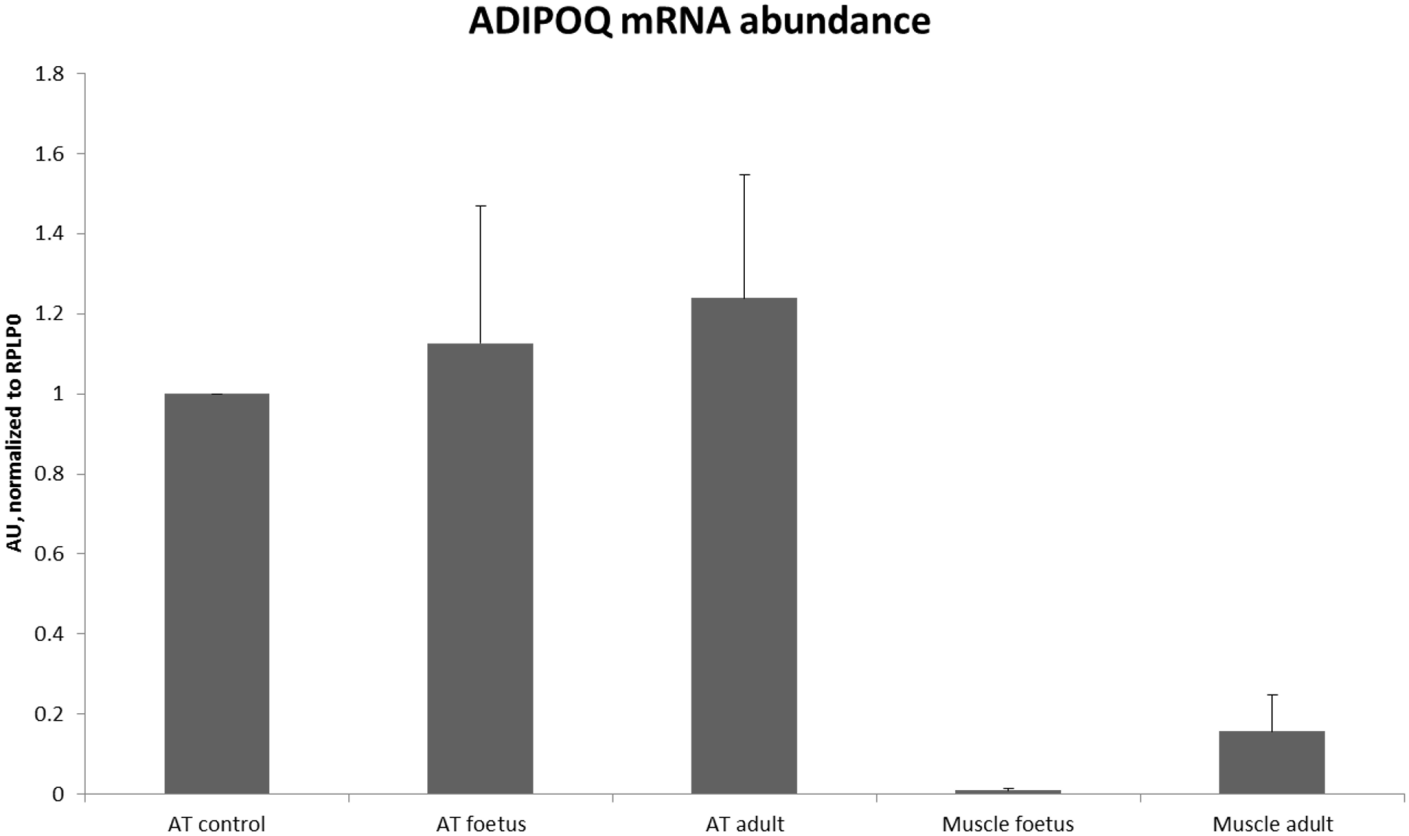
mRNA abundance of adiponectin (ADIPOQ) in bovine AT and muscle. The abundance of ADIPOQ was normalized to the mRNA abundance of ribosomal protein P0 (RPLP0). Results are ΔΔ CT for foetal and adult AT or muscle samples, relatively to a control sample that is an adult AT. PCR were carried out as previously described [62].

The 46 proteins were annotated by enriched GO terms (*p*-value < 0.001 corrected by the BH test) related to apoptosis (negative regulation of apoptotic process, apoptotic process, and regulation of apoptotic process), the progression of cell cycle (DNA damage response, signal transduction by p53 class mediator resulting in cell cycle arrest, regulation of ubiquitin-protein ligase activity involved in mitotic cell cycle, anaphase-promoting complex-dependent proteasomal ubiquitin-dependent protein catabolic process, and G1/S transition of mitotic cell cycle) metabolic processes (regulation of cellular amino acid metabolic process, respiratory electron transport chain, glucose metabolic process, and protein polyubiquitination), the regulation of cytokine- or interleukin-mediated signaling pathways (negative regulation of tumor necrosis factor-mediated signalling pathway and negative regulation of interleukin-8 secretion). Six of these 46 proteins were predicted and confirmed by GO terms and TargetP as being secreted because of a signal peptide (Table 5). As previously discussed in sections 2.1 and 2.2, all of them were reported in the bovine plasma [48]. The identification of ADIPOQ in both bovine foetal tissues, and especially in the muscle, is surprising because intramuscular adipocytes believed to synthetize adiponectin [59], were histologically characterised after birth in bovine [2]. Thus, to question the synthesis and secretion or the uptake of adiponectin by the foetal muscle we assayed its mRNA abundance (Fig 7). The very low abundance of ADIPOQ mRNA in foetal muscle, when compared to adult or foetal AT, suggests that ADIPOQ is rather taken up than synthetized by foetal muscle. The role of the adipose ADIPOQ [4] within muscle remained to be studied during the bovine foetal growth. ProteINSIDE had also predicted 32 potentially secreted proteins without signal peptide. However, only 5 proteins were both confirmed by GO terms and TargetP (CAPZA2, TPI1, ARHGDIA, HNRNPK, and EIF5A), and a last protein was also confirmed by Subcellular location (PRD6X). As described in section 2.1 and 2.2 some of these proteins are known to be secreted by AT and skeletal muscle in adult (PRD6X, TPI1, ARHGDIA, and EIF5A).

**Fig 8:**
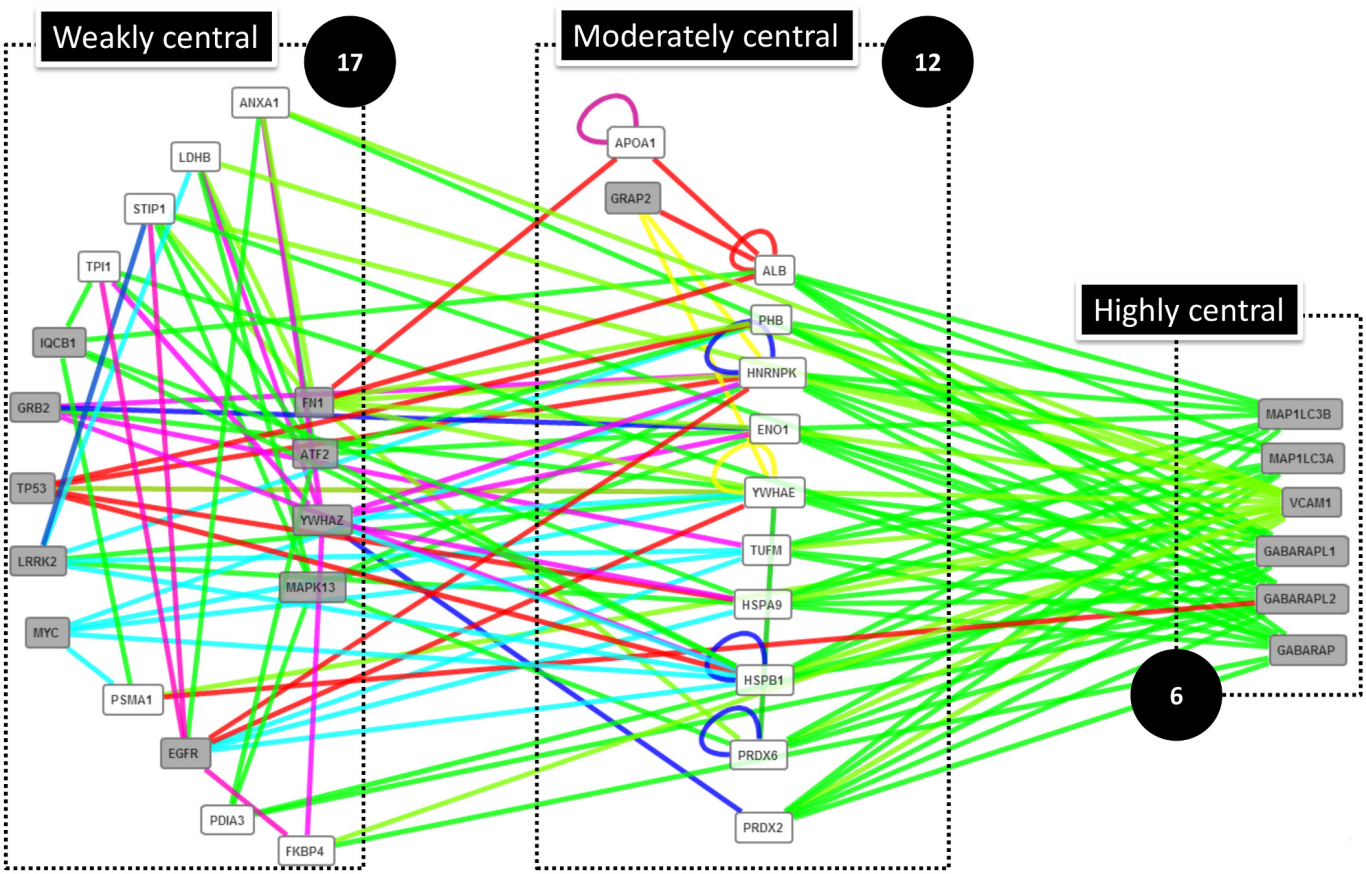
Extended PPi network that includes proteins identified both in foetal AT and muscle. ProteINSIDE built a network between the 46 proteins identified in AT and muscle, and proteins outside of the dataset and know to interact with them in Human species. We filtered and sorted the network using high values of betweenness (an average of 10000) and closeness centralities (an average of 0,318). We have identified 6 proteins that were highly central in the dataset (linked with the maximum of proteins and pathways), 12 proteins were moderately central (engaged in the maximum of pathways but not necessary with many proteins), and 17 proteins were more weakly central (less linked with the maximum of proteins and less engaged in pathways, but central on the network). White boxes indicate proteins that are from the bovine dataset and grey boxes indicate proteins that are external to the dataset.

Among the 46 common proteins, 23 were linked by 40 interactions and grouped in 2 subnetworks of proteins related to muscle proteasome and mitochondrial metabolism (already described in Fig 5C and 5A, respectively). In order to identify new putative key proteins for the foetal growth of AT and muscle, we have extended the network by including proteins known to interact with the 46 proteins in Human. The extended network linked 1063 proteins (41 from the dataset) by 1400 interactions. By using high values of betweenness and closeness centralities, we proposed 35 proteins (18 from dataset; Fig 8) as being central in the extended network. Among them, 6 proteins (external of the dataset) were highly central: VCAM1, GABARAPL1, GABARAPL2, GABARAP, MAP1LC3A, and MAP1LC3B. The presence of these proteins or their mRNA in adipose and muscular cells was reported for VCAM1 [63,64], GABARAPL1 [65,66], GABARAPL2 [64,67], GABARAP [64,68], MAP1LC3A [64,69], and MAP1LC3B [64,70].

VCAM1 also named CD106 is a cell surface marker of mesenchymal stem cells found in AT [71] and muscle [72]. The presence of VCAM1 in foetal bovine tissue remains to be shown but should indicate that mesenchymal stem cells contribute to the post-embryonic growth of AT and muscle as already proposed [2]. GABARAPL1, GABARAPL2, GABARAP, MAP1LC3A, and MAP1LC3B are members of Atg8 family in higher eukaryotes and involved in autophagy, a major catabolic pathway that impacts several cellular pathways such as cell survival, differentiation, tumorigenesis… The centrality of these autophagy-related proteins in the extended network suggests that autophagy plays a role in the differentiation and growth of adipose and muscle tissues in bovine foetuses. This remains to be studied. However, this hypothesis is supported by recent *in vitro* data that demonstrate that autophagy is increased and required for the differentiation of C2C12 myoblasts [73] and 3T3-L1 preadipocytes [74] probably to sustain the significant morphological and biochemical remodelling of differentiating cells. Moreover *in vivo*, the deletion of an autophagy-related gene in mice impairs foetal adipogenesis, consequently pups have only 15% of differentiated adipocytes in subcutaneous AT when compared to wild-type counterparts [75]. The deletion of an autophagy-related gene in Myf5+ progenitor cells impairs brown AT differentiation and function, muscle differentiation, reduces muscle mass, and leads to glucose intolerance in mice [76]. The study of the expression of autophagy-related genes by comparison with some markers of adipogenesis and myogenesis or secreted factors that we have identified, such as adiponectin, should help to understand the growth of AT relatively to muscle in bovine foetuses.

## Conclusions

Using a list of genes or proteins, ProteINSIDE proceeds to a fast overview of biological information for ID of the uploaded dataset, a functional annotation according to GO, a prediction of secreted proteins, and a simple and interactive view of known and experimentally proven PPi. The similar or better reliability of results produced by ProteINSIDE was demonstrated by a bench test involving others available resources. Moreover, we have verified the biological relevance of the results by comparison with our previous analyses. Lastly, we have used ProteINSIDE to propose new hypotheses of research that should help to better understand the growth of AT and muscle in bovine. Among the new hypotheses of research that deserved to be investigated, we have focused on an uptake of adiponectin by the foetal muscle and the role of autophagy on the ontogenesis of AT and muscle. To go further, ProteINSIDE can easily be upgraded to broaden the range of species (as other farm species: e.g. pig, chicken…) by an update of the database and the workflow.

## Supporting Information

S1 Table Lists of 1000 proteins ID from the 6 species used to test ProteINSIDE’s performances. (XLSX)

S2 Table Lists adipose and muscle tissues proteins from Taga et al. [4], and Chaze et al. [3,5]. (XLSX)
